# When Love Hurts – Mental and Physical Health Among Recently Divorced Danes

**DOI:** 10.3389/fpsyg.2020.578083

**Published:** 2020-11-30

**Authors:** Søren Sander, Jenna Marie Strizzi, Camilla S. Øverup, Ana Cipric, Gert Martin Hald

**Affiliations:** Department of Public Health, University of Copenhagen, Copenhagen, Denmark

**Keywords:** divorce, divorce intervention, mental health, physical health, Danes

## Abstract

The last decades of research have consistently found strong associations between divorce and adverse health outcomes among adults. However, limitations of a majority of this research include (a) lack of “real-time” research, i.e., research employing data collected very shortly after juridical divorce where little or no separation periods have been effectuated, (b) research employing thoroughly validated and population-normed measures against which study results can be compared, and (c) research including a comprehensive array of previously researched sociodemographic- and divorce-related variables. The current cross-sectional study, including 1,856 recently divorced Danes, was designed to bridge these important gaps in the literature. Mental and physical health were measured using the Short Form 36 (SF-36)-2. Analyses included correlational analyses, *t-*test comparisons, and hierarchical multiple regression analyses. The study found that the health-related quality of life of Danish divorcees was significantly worse than the comparative background population immediately following divorce. Across gender, higher levels of divorce conflict were found to predict worse mental health, and worse physical health for women, even when controlling for other socio-demographic variables and divorce characteristics. Among men, lower age and higher income predicted better physical health, while more children, more previous divorces, participant divorce initiation, new partner status, and lower levels of divorce conflict predicted better mental health. Among women, higher income, fewer previous divorces, new partner status, and lower levels of divorce conflict predicted better physical health while higher income, participant divorce initiation, new partner status, and lower levels of divorce conflict predicted better mental health. The findings underscore the relevance of providing assistance to divorcees who experience higher levels of divorce conflict immediately following divorce, in seeking to reduce potential long-term negative health effects of divorce.

## Introduction

The last 20 years of research have consistently found strong associations between divorce and adverse health outcomes among adults. Generally, divorcees report poorer physical and mental health and more symptoms of stress, anxiety, depression, and social isolation than the general population (Amato, 2000, 2010; Kessing et al., 2003; Hewitt and Turrell, 2011; Hewitt et al., 2012; Hald et al., 2020b). Furthermore, divorce is associated with more frequent hospitalization (Nielsen et al., 2014), substance use (Waite et al., 2009), higher suicide rates (Kposowa, 2000), lower levels of psychological well-being (Bracke et al., 2010; Colman et al., 2012), and greater overall mortality risk (Kposowa, 2000; Sbarra and Nietert, 2009). However, four limitations relate to a significant part of this research.

First, often studies include only one or two health-related outcomes per study (e.g., stress and/or depression) (e.g., Lindström, 2009; Hewitt et al., 2012; Knöpfli et al., 2016). While this is important in mapping out specific effects of divorce, it limits the ability to gain insight into more comprehensive physical and mental health profiles among divorce populations. These could be important for more accurate and comprehensive assessments and profiling of the effects of divorce on health. Second, most countries in the world require separation periods before juridical divorce is granted. This means that divorce studies able to employ “real-time” research are scarce and there has been a call for such studies (e.g., Thuen, 2001; Cipric et al., 2020). The concept of “real-time” research usually refers to the collection of data among divorcees with little or no separation periods before formal juridical divorce (Hald et al., 2020a). When studying health effects of divorce, this may be especially important since many health outcomes related to divorce may be sensitive to a “time heals effect,” whereby negative effects of divorce naturally decline over time (Amato, 2010; Sander et al., 2020). Therefore, current research on adverse health effects of divorce may, in fact, underestimate negative health effects of divorce as data have often been collected after a divorce that was preceded by significant periods of separation and thus is likely to be subject to the “time heals effect” (Sander et al., 2020). Third, studies employing thoroughly validated and population-normed measures are few. Validated measures are needed for accurate assessment of the health outcomes studied. However, these assessments may benefit from contextualization by having background population norms against which the results can be directly compared. This allows for more direct insights into the degree to which divorcees may differ from background population norms and thus the relative impact of the divorce on health. Fourth, studies are needed that include a more comprehensive array of previously researched sociodemographic- and divorce-related predictor or explanatory variables of mental and physical health. This would allow for a more thorough assessment of the individual and combined effect of these variables on mental and physical health. The current study was designed to bridge these four important gaps in health research related to divorce.

Divorce theory and divorce research suggest that there are sociodemographic variables and divorce-related characteristics that may moderate the effects of divorce on mental and physical health. Theoretically, Amato’s Divorce-Stress-Readjustment perspective (DSR; Amato, 2000) suggests that adverse effects of divorce depend on a number of risk and protective factors experienced during and following the divorce process. Examples of risk factors include lower standards of living, loss of benefits associated with marriage, and conflict with the former partner, whereas examples of protective factors include having a new romantic partner, adequate income, and holding positive views about the divorce. According to the DSR, it is the interplay between risk and protective factors that may be important in determining the effects of divorce on mental and physical health (Amato, 2010).

From an empirical perspective, studies suggest that lower socioeconomic status, being unemployed, lower levels of education, and lower family income (Barrett, 2000; Simon, 2002; Symoens et al., 2013b) are associated with lower mental and physical health following divorce. In addition, younger age has been found to be associated with lower mental health following divorce (Bulloch et al., 2017). In relation to divorce characteristics, mutual divorce agreement initiation (Weiss, 1976; Gray and Silver, 1990; Wang and Amato, 2000; Sweeney and Horwitz, 2001; Sakraida, 2008; Cohen and Finzi-Dottan, 2012; Symoens et al., 2013a), having a new partner (Mastekaasa, 1994; Amato, 2000; Øygard, 2004; Blekesaune, 2008; Kulik and Heine-Cohen, 2011; Symoens et al., 2013b; Symoens et al., 2014) and lower levels of divorce-related conflict (Symoens et al., 2014; Petren et al., 2017) have been found to be associated with better mental and physical health. Both empirically and from an applied point of view, divorce conflict has been found to adversely affect or accelerate declines in mental health among divorcees. While the cross-sectional nature of the current study does not allow for investigation of the impact of divorce conflict on mental health over time, it does allow for an independent assessment of the explanatory value of divorce conflict on mental health, accounting for basic sociodemographic variables and other divorce-related characteristics. Compared with previous research, this allows for a more thorough and “independent” investigation of divorce conflict on mental health immediately following divorce.

The current study took place in Denmark, providing a unique perspective on divorce and divorce-related processes. First, in Denmark, there is high societal acceptance of divorce (Uggla and Andersson, 2018), and in general, divorce is not associated with societal stigma, as it is in many other parts of the world. Second, Denmark is a country with high levels of equality, both in terms of gender equality (European Institute for Gender Equality, 2018) and income equality (OECD, 2018). As such, Denmark offers a unique context in which to study whether sociodemographic and divorce-related factors predict post-divorce mental and physical health.

Based on the above, the current study sought to investigate mental and physical health among recently divorced Danes using a well-known, comprehensive, and population-normed mental and physical health measure. Further, the study sought to examine the explanatory value of a comprehensive array of previously identified sociodemographic variables and divorce-related characteristics on overall mental and physical health. Finally, the study sought to compare overall mental and physical health to relevant population norms. Accordingly, the following two research questions and one study hypothesis guided the study investigation:

*RQ1:* What is the mental and physical health among recently divorced individuals and how does it compare to population norms?
*RQ2:* What is the explanatory value of sociodemographic variables (i.e., age, number of children, income, education) and divorce-related characteristics (i.e., marriage duration, number of previous divorces, divorce initiator status, new partner status, and divorce conflict) on overall mental and physical health among recently divorced individuals?
*H1:* Divorce conflict will significantly add to the explanatory value of mental health after accounting for basic sociodemographic variables (i.e., age, number of children, income, education) and divorce-related characteristics (i.e., marriage duration, number of previous divorces, divorce initiator status, and new partner status).

## Materials and Methods

### Participants

The study sample comprised 1,856 participants of which 66% were women. The average age of women was 44.65 years (*SD* = 8.34), while for men, it was 46.66 years (*SD* = 9.31). The majority of participants had at least a medium educational level and earned at least the national average salary (see Table 1). The majority of the sample (88.3%) were parents, with an average of 1.88 (*SD* = 0.99) children per participant. The average marriage duration for men was 12.22 years (*SD* = 8.11) and for women 13.0 (*SD* = 7.98), and for approximately 88% of the sample, this was their first divorce. A majority of women (52%) reported to have initiated the divorce, with 29% of men reporting to be divorce initiators. The majority of both male and female participants did not have new partners following their divorce (65% men, 64% women). The mean legal divorce duration before survey completion was 4.47 days (*SD* = 6.97) for men and 5.23 (*SD* = 7.66) days for women. Of note, there were some gender differences in sociodemographic and divorce-related characteristics. Specifically, compared to men, women were younger, had been married slightly longer, were more highly educated, earned less than men, had initiated the divorce more often, and had a different partner status than men [age (*t*(1854) = 4.74, *p* < 0.001); duration of marriage (*t*(1854) = −1.972, *p* = 0.049); education (χ^2^ = 32.61, *p* < 0.001); income (χ^2^ = 107.41, *p* < 0.001); initiator status (χ^2^ = 90.50, *p* < 0.001); new partner (χ^2^ = 14.82, *p* = 0.002)].

**TABLE 1:** Participant demographics (*N* = 1,856).

**Variable**	**Men**	**Women**
Age, years, mean (*SD*)	46.66 (9.31)	44.65 (8.34)**
**Number of children, %**		
0	13.3	11.0
1	15.2	15.8
2	49.3	49.7
3	19.1	19.6
4 or more	3.1	3.9
**Education level, %**		
Low level of education	43.9	32.5**
Medium level of education	28.8	41.5
High level of education	27.2	26.0
**Income, %**		
Below national average salary	26.7	47.7**
National average	47.0	41.8
Above national average salary	26.3	10.8
Marriage length, mean (*SD*)	12.22 (8.11)	13.0 (7.98)*
Total divorce duration in days, mean (SD)^a^	4.47 (6.97)	5.23 (7.66)
**Number of times divorced, %**		
One time	86.7	88.2
Two times	10.7	10.1
Three times	1.9	1.5
More than three times	0.6	0.2
**Initiative divorce, %**		
Participant	28.5	51.8**
Mutual agreement	19.2	13.2
Former spouse	52.3	35.0
**New partner, %**		
Both have new partners	3.6	5.3*
Neither have new partners	64.7	63.7
Participant does, former spouse does not	13.5	8.7
Participant does not, former spouse does	18.3	22.3
Divorce Conflict Scale Scores, mean (SD)	13.28 (4.92)	13.97 (4.97)*

^*a*^Legal divorce duration was calculated in days from the legal divorce date to survey response date. *p < 0.05. **p < 0.001.

Data on all people who divorced in Denmark during the study period were obtained from Statistics Denmark and compared to the study sample. The study sample was found to be representative in terms of age, income, and marriage duration (*p* > 0.05). There were statistically significant differences between participants and the comparison population in terms of gender (more women participated: χ^2^ = 208.45, *p* < 0.001), educational attainment (study participants were more highly educated: χ^2^ = 1135.23, *p* < 0.001), and the number of previous divorces [participants had on average fewer previous divorces than the average Danish divorcee: *t*(1855) = −8.47, *p* < 0.001].

### Procedure

During the study period (January 2016 to January 2018), those seeking divorce in Denmark initiated formal legal divorce and separation procedures by submitting an application to the Danish State Administration (DSA). Legal divorce was granted immediately when there was a mutual agreement to the marital dissolution. However, if there was disagreement regarding the divorce itself or its terms, a 6-month separation period was instituted, after which divorce was granted even in the absence of mutual agreement. The DSA reports that approximately 30% of couples underwent the 6-month separation period. The average processing time required by the DSA to issue divorce decrees was 2–3 weeks.

Invitations to the present study were sent by the DSA along with the divorce decree. The invitation letter described the 12-month Randomized Controlled Trial intervention study entitled “Cooperation after Divorce” that sought to investigate the effects of a digital intervention platform called “Cooperation after Divorce (CAD)” on divorcees’ mental and physical health. As the DSA sent out invitations, we were unable to send re-invitations to those who did not respond to the initial invitation sent out by the DSA. Those who completed the baseline survey received invitations from the intervention platform to complete surveys at 3, 6, and 12 months; for each of these time points, two reminder e-mails were sent out, one after 3 days and one after 14 days, if no response had been provided.

Cooperation after Divorce covers three main areas: (1) the divorce, (2) children, and (3) cooperation following divorce, employing 17 learning modules delivered through an online platform. This paper reports only the baseline results of the study, therefore, please also see Hald et al. (2020a) for a more thorough description of the CAD platform. The letter also described the procedure for participation, which consisted of clicking on a web-link in the invitation letter, provide informed consent, and respond to the baseline questionnaire anonymously. The research received approval from the Danish Data Protection Agency and was exempt from further ethical evaluations following the rules and regulations as set forth by the Scientific Ethical Committees of Denmark.

The exact response rate is not possible to report because the DSA could not provide the precise number of study invitations sent during the study period. There were 32,487 legal divorces in Denmark during the RCT enrollment period; however, it is unknown whether all individuals who divorced received an invitation along with their divorce decree. In total, 1,882 people began the study and due to impossible or invalid responses, 26 were excluded (i.e., those who did not report gender, reported to be married less than 1 day, or to have married the same year as they were born). Thus, 1,856 participants were included in the final analytical study sample.

### Measures

#### Sociodemographic Variables

(a) Age at divorce was measured in years and months. (b) Sexual identity was determined by answering: “Are you a man or a woman?” with the response options: 1 = “Man” 2 = “Woman.” (c) Education level was assessed by answering: “What is the highest education you have completed?” with the following response options: 1 = “low level of education” (e.g., primary school, high school, business high school, vocational education), 2 = “medium level of education” (e.g., medium-length tertiary education, bachelor’s degree), and 3 = “high level of education” (e.g., master’s degree or higher). (d) Income was measured with the question “What is your monthly income before tax?” in Danish Crowns (1 USD = 6.35 DKK). The response options were: 1 = “Below 10,000DKK,” 2 = “10–20,000DKK,” 3 = “20–30,000DKK,” 4 = “30–40,000DKK,” 5 = “40–50,000DKK,” 6 = “50–60,000DKK,” 7 = “60–70,000DKK,” 8 = “70–80,000DKK,” 9 = “More than 80,000DKK.” These categories were reduced for descriptive purposes for Table 1 so that 1–3 = “Below average,” 2–4 = “Average,” 5+= “Above average”; however, in all analyses the original scale was used. (e) The number of children was obtained by asking how many children participants had from 0 to 8.

#### Divorce-Related Variables

(a) Marriage duration was calculated in years and months from marriage date to divorce date; (b) legal divorce duration was calculated in days from the legal divorce date to survey response date; (c) number of divorces was obtained by asking, “How many time have you divorced?” with response options including 1 = “One time,” 2 = “Two times,” 3 = “Three times,” and 4 = “More than three times”; (e) divorce initiator status was ascertained with the question “Who initiated your divorce” and 1 = “Me,” 2 = “Mostly me,” 3 = “We mutually agreed,” 4 = “Mostly my former spouse,” 5 = “My former spouse,” 6 “Not sure.” Initiator status responses were reduced so that 1–2 = “Me,” 3 = “We mutually agreed,” 4–5 = “My former spouse,” and 6 = “System missing” [only seven participants (0.4%) responded “not sure”]; (f) New partner status was obtained with the question “Do you or your ex have a new partner?” with the following response options: 1 = “Yes, we both have a new partner,” 2 = “No, none of us have a new partner,” 3 = “I have a new partner, but not my ex,” 4 = “My ex has a new partner, but not me”; (g) Divorce conflict was assessed employing the six-item self-report Divorce Conflict Scale (DCS). The DCS measures six dimensions of divorce-related conflict: communication, co-parenting, global assessment of former spouse, negative and pervasive negative exchanges and hostile, insecure emotional environment, and self-perceived conflict (Hald et al., 2020d). The internal consistency of the DCS scale was high (α = 0.88).

#### Physical and Mental Health

The second version of the Short Form 36 (SF-36) Health Assessment was used for the core outcomes of this study. The SF-36 is a 36-item self-report measure that is a widely used instrument to assess health-related quality of life over the previous 4 weeks among general populations and diverse patient groups (Maruish, 2011). The instrument includes the following eight domains which are measured using 35 items: physical functioning, role physical (role participation with physical health problems), bodily pain, general health, vitality, social functioning, role-emotional (role participation with emotional health problems), and mental health. The final item is not included in the domains subscales and addresses self-evaluation health transition. The responses are given with a Likert scale or a yes/no format. Domain scores are reported in 0–100 transformed scores and *t*-scores that are calculated from the raw scores and higher scores indicate better health status (see Maruish, 2011 for more information). The physical health and mental health summary variables are calculated using all eight health domains based on their relative factor analytical weights. Many language versions of the SF-36 exist and the instrument has been determined to be a valid and reliable instrument for a wide range of populations (Bjorner et al., 1998; Maruish, 2011). In this study, all of the eight health scales demonstrated high internal consistency (Cronbach’s α = 0.85–0.93).

### Data Analyses

Missing data were less than 5% for all variables in the present paper, which is below the proportion of missingness that may bias results (Schafer, 1999; Bennett, 2001; Dong and Peng, 2013). Thus, the data were omitted “listwise” in analyses. For the legal divorce duration variable, outliers were changed to missing values using the moderately conservative ± 2.5 times the median absolute deviation (MAD) threshold, as recommended by Leys et al. (2013). To assess gender differences, sociodemographic and divorce-related characteristics were compared using two-sample *t*-tests and chi-square tests.

Prior to any other data analyses, a rake weight was constructed and applied to the data. The rake weight was based on gender, education, and previous number of divorces and adjusted for sample representativeness (see section “Participants”). When constructing rake weights, a set of variables for which the distribution is known are chosen, and the statistical program creates weights for each case until the sample distribution aligns with the population for those variables. The resultant weight was applied to the data. Thus, all following data analyses (correlations, comparisons to norms, cut-off score comparison, and hierarchical regressions) reflect results with the weight applied.

One-sample *t*-tests were employed to compare our sample with the available Danish normative data from the Danish SF-36 user’s manual, which comprise a random population sample of 4,080 Danish adults (52% women) from the SF-36 Health Assessment Danish Manual study (for more information regarding this normative population sample, see also Bjorner et al., 1998). For comparisons, the SF-36 0–100 transformed scale scores were used.

Pearson correlation analyses were used for assessing bivariate correlations between variables. Hierarchical multiple regression analyses were used to assess the independent contribution to the explanation of the variance SF-36 physical and mental health summary *t*-scores. In a first step, age, number of children, income, and education were entered as predictors; in a second step, marriage duration, number of previous divorces, divorce initiator status, and new partner status were entered as predictors. DCS scores were entered as a predictor in the third step. This approach allows for an assessment of the unique contributions of sets of variables (i.e., demographics and divorce-related variables), and specifically, allows for an assessment of the unique contribution of divorce conflict, beyond the contribution of demographics and divorce-related factors.

## Results

When compared with Danish normative data, male participants reported lower role physical scores [*t*(878) = −9.38, *p* < 0.001, *d* = 0.32], worse general health [*t*(878) = −5.66, *p* < 0.001, Cohen’s *d* = 0.19], lower vitality [*t*(875) = −31.88, *p* < 0.001, Cohen’s *d* = 1.08], decreased social functioning [*t*(878) = −23.51, *p* < 0.001, Cohen’s *d* = 0.79], lower role emotional scores [*t*(878) = −25.63, *p* < 0.001, Cohen’s *d* = 0.87], and worse mental health [*t*(875) = −40.79, *p* < 0.001, Cohen’s *d* = 1.38], but better physical functioning [*t*(879) = 6.66, *p* < 0.001, Cohen’s *d* = 0.23] and lower levels of bodily pain [*t*(878) = 2.34, *p* = 0.020, Cohen’s *d* = 0.08], than the Danish normative male population.

**FIGURE 1:**
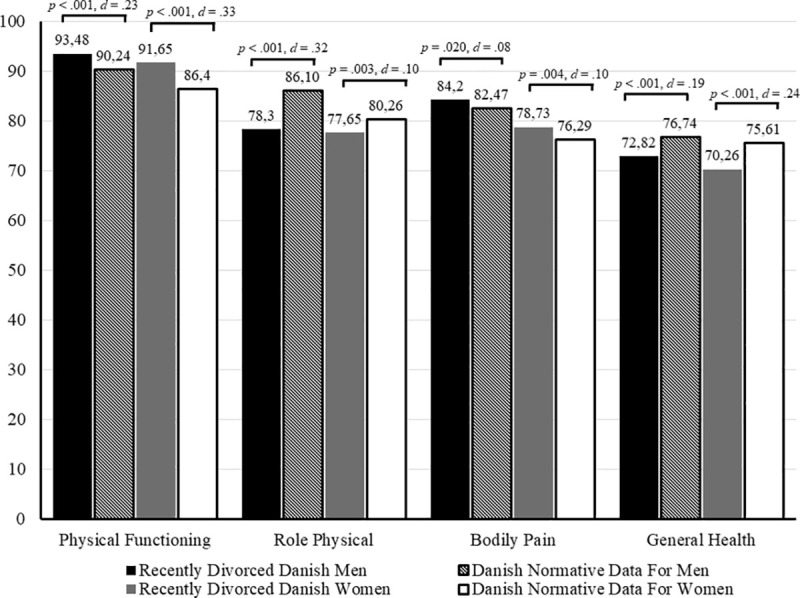
SF-36 physical health domain means compared to normative data.

**FIGURE 2:**
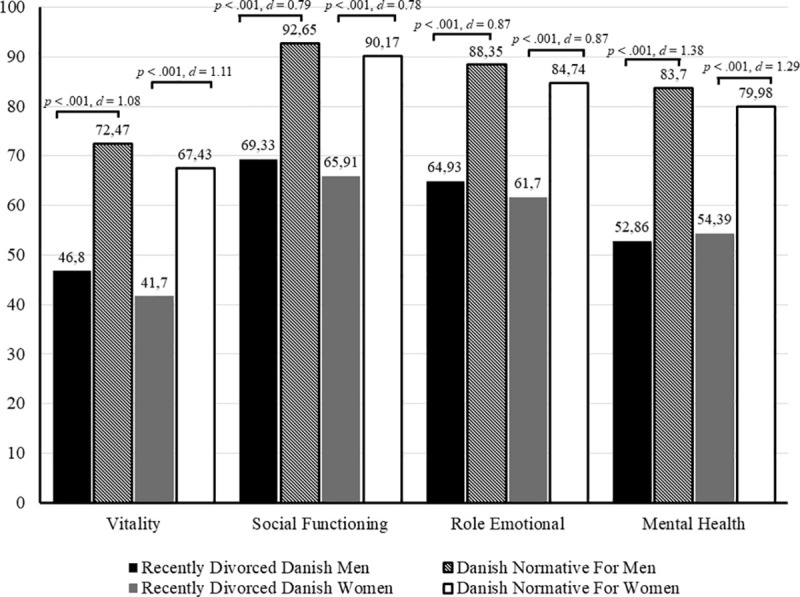
SF-36 mental health domain means compared to normative data.

Statistically significant differences were found on the SF-36 domains for women. Compared with the Danish normative female population, female participants reported lower role physical scores [*t*(880) = −3.00, *p* = 0.003, *d* = 0.10], worse general health [*t*(883) = −7.25, *p* < 0.001, Cohen’s *d* = 0.24], lower vitality [*t*(878) = −33.00, *p* < 0.001, Cohen’s *d* = 1.11], lower social functioning scores [*t*(880) = −23.19, *p* < 0.001, Cohen’s *d* = 0.78], decreased role emotional capacity [*t*(880) = −25.86, *p* < 0.001, Cohen’s *d* = 0.87], and worse mental health [*t*(878) = −38.31, *p* < 0.001, Cohen’s *d* = 1.29], but better physical functioning [*t*(883) = 9.94, *p* < 0.001, Cohen’s *d* = 0.33] and lower levels of bodily pain [*t*(880) = 2.92, *p* = 0.004, Cohen’s *d* = 0.10] (see Figures 1, 2).

Comparison cut-off scores were created such that those with *t*-scores below 44 were categorized as poor functioning, those with *t*-scores between 44 and 56 (i.e., average) were categorized as normal functioning, and those with *t*-scores above 56 (i.e., above) were categorized as superior functioning. The comparisons revealed that for the intervention group, 8.3% fell below the cut-score on physical health (normal = 23.8% and superior = 68%) and 73.6% fell below the cut-score on mental health (normal = 19.9% and superior = 6.6%). Similarly, for the control group, 8.0% fell below the cut-score on physical health (normal = 22.5% and superior = 69.5%) and 72.6% fell below the cut-score on mental health (normal = 23.8% and superior = 3.6%).

Among men, bivariate correlation analyses demonstrated that lower age, higher income, higher education, shorter duration marriages, fewer previous divorces, and lower mental health scores were significantly associated with better physical health (*p* < 0.05). Among women, lower age, higher income, higher educational level, fewer previous divorces, new partner status, lower divorce conflict, and lower mental health scores were significantly associated with better physical health (*p* < 0.05). Among men, higher age, longer marriage duration, more previous divorces, initiator and new partner status, and lower divorce conflict scores were significantly associated with better mental health, while for women higher income, fewer previous divorces, initiator status, and lower divorce conflict scores were significantly associated with better mental health (*p* < 0.05; see also Table 2).

**TABLE 2:** Correlations among sociodemographic variables, divorce conflict scale scores, physical and mental health summary scores (*N* = 1856, men *n* = 617, women *n* = 1239).

	**Variables**	**1**	**2**	**3**	**4**	**5**	**6**	**7**	**8**	**9**	**10**	**11**
1	Age	–	0.026	−0.094**	0.080*	0.560**	0.354**	0.048	0.104**	0.155**	−0.097**	−0.022
2	Number of children	−0.026	–	0.011	−0.064	0.297**	−0.140**	−0.092**	0.001	0.022	0.037	0.033
3	Education	0.013	0.032	–	0.331**	−0.072*	−0.103**	−0.049	−0.023	−0.047	0.116**	0.046
4	Income	−0.006	0.090**	0.304**	–	0.053	−0.012	−0.013	0.082*	−0.051	0.214**	0.114**
5	Marriage duration	0.459**	0.204**	0.037	0.167**	–	−0.193**	−0.027	0.145**	0.096**	−0.011	0.033
6	Number of prev. divorces	0.498**	−0.121**	−0.040	−0.159**	−0.184**	–	0.050	−0.023	0.102**	−0.131**	−0.080*
7	Initiator status	−0.116**	0.031	−0.075*	−0.133**	−0.067*	−0.052	–	0.199**	0.048	0.058	−0.215**
8	New partner status	−0.109**	0.116**	−0.052	−0.039	−0.121**	−0.089**	0.057	–	0.196**	0.087**	−0.020
9	Divorce Conflict Scale	−0.019	0.027	−0.050	−0.071*	−0.094**	−0.012	−0.138**	0.142**	–	−0.078*	−0.144**
10	Physical Health Summary	−0.260**	0.019	0.116**	0.240**	−0.121**	−0.159**	0.008	0.041	−0.056	–	−0.095**
11	Mental Health Summary	0.256**	0.047	0.043	0.040	0.127**	0.271**	−0.200**	−0.171**	−0.131**	−0.165**	–

*p < 0.05. **p < 0.01. Correlations for women are above the diagonal and correlations for men are below the diagonal.

Force enter hierarchical multiple regression analyses were used to assess whether socio-demographic and divorce characteristics predicted mental and physical health and whether divorce conflict added to the explanatory value of mental health after controlling for sociodemographic variables and divorce characteristics. The first step of the analyses included the sociodemographic variables of age, number of children, income, and education, and the second step included the divorce-related variables of marriage duration, number of previous divorces, divorce initiator status, and new partner status, while the third and final step included divorce conflict. The variables (Step 3) explained 14.6% of the variance of the physical health summary scores for men [*F*(12,875) = 12.33, *p* < 0.001, *R*^2^ = 0.146] and 8.8% for women [*F*(12,878) = 6.96, *p* < 0.001, *R*^2^ = 0.088]. Among men, lower age and higher income significantly added to the prediction of better physical health (*p* < 0.05). Among women, higher income, fewer previous divorces, new partner status, and lower divorce conflict added to the prediction of better physical health (*p* < 0.05) (see also Table 3).

**TABLE 3:** Multiple regression analyses predicting SF-36 physical health summary *t*-scores.

**Variable**	***B***	***SE B***	**β**	***B***	***SE B***	**β**	***B***	***SE B***	**β**
**Men**									
Age	−0.225**	0.027	−0.261	−0.198**	0.042	−0.230	−0.194**	0.042	−0.225
Number of children	0.020	0.274	0.002	0.176	0.286	0.020	0.221	0.286	0.026
Education	0.683	0.417	0.054	0.729	0.416	0.058	0.702	0.416	0.056
Income	2.600**	0.383	0.225	2.733**	0.393	0.236	2.686**	0.393	0.232
Duration of marriage				−0.057	0.048	−0.053	−0.065	0.048	−0.062
Number of times divorced				−0.108	0.601	−0.008	−0.162	0.601	−0.012
Initiator Status: Participant vs Former Spouse				−0.507	0.656	−0.031	−0.769	0.670	−0.047
Initiator Status: Participant vs Mutual Agreement				−1.173	0.827	−0.056	−1.546	0.850	−0.074
New Partner Status: Both vs neither				0.837	1.368	0.049	0.849	1.366	0.050
New Partner Status: Both vs Participant Yes, Ex No				−1.766	1.539	−0.071	−1.749	1.537	−0.070
New Partner Status: Both vs Participant No, Ex Yes				1.384	1.462	0.068	1.543	1.462	0.076
Divorce Conflict							−0.103	0.055	−0.062
*R*		0.36			0.38			0.38	
Adjusted *R*^2^		0.12			0.13			0.13	
*F*		31.99**			13.10**			12.33**	
Change *R*^2^					0.02			0.003	
*F* Change *R*^2^					2.14*			3.47	
**Women**									
Age	−0.117**	0.034	−0.113	−0.088	0.051	−0.085	−0.081	0.051	−0.079
Number of children	0.536	0.299	0.059	0.498	0.319	0.055	0.505	0.318	0.055
Education	0.451	0.454	0.035	0.441	0.453	0.034	0.432	0.452	0.033
Income	3.001**	0.487	0.216	2.930**	0.487	0.211	2.859**	0.487	0.206
Duration of marriage				−0.009	0.053	−0.009	−0.008	0.053	−0.007
Number of times divorced				−1.808*	0.760	−0.096	−1.711*	0.760	−0.091
Initiator Status: Participant vs Former Spouse				1.098	0.666	0.059	1.094	0.664	0.059
Initiator Status: Participant vs Mutual Agreement				−0.813	0.904	−0.031	−1.086	0.911	−0.041
New Partner Status: Both vs neither				1.637	1.342	0.089	1.511	1.341	0.082
New Partner Status: Both vs Participant Yes, Ex No				1.432	1.633	0.045	1.340	1.630	0.042
New Partner Status: Both vs Participant No, Ex Yes				2.728	1.455	0.129	2.937*	1.456	0.139
Divorce Conflict							−0.133*	0.062	−0.073
*R*		0.25			0.29			0.30	
Adjusted *R*^2^		0.06			0.07			0.08	
*F*		14.76**			7.15**			6.96**	
Change *R*^2^					0.02			0.005	
*F* Change *R*^2^					2.69*			4.52*	

*p < 0.05. **p < 0.001. When analyses were run with number of previous divorces coded as “1/2/3 or more,” the pattern of results remained the same. Moreover, when analyses were run with number of children coded as “1/2/3 or more,” the pattern of results also remained the same.

For mental health, sociodemographic and divorce-related variables, as well as divorce conflict (Step 3) accounted for 19.3% of the explained variance among men [*F*(12,875) = 17.15, *p* < 0.001, *R*^2^ = 0.193] and 9.9% among women [*F*(12,878) = 7.89, *p* < 0.001, *R*^2^ = 0.099]. Factors that significantly added to the prediction of better mental health for men were more children, more previous divorces, participant divorce initiation, new partner status, and lower divorce conflict, while for women, higher income, participant divorce initiation, new partner status, and lower divorce conflict significantly added to the prediction of better mental health.

Regarding the study hypothesis, among both men and women, divorce conflict was found to significantly add to the explanation of mental health after controlling for basic sociodemographic variables and divorce characteristics (see also Table 4).

**TABLE 4:** Multiple regression analyses predicting SF-36 mental health summary *t*-scores.

**Variable**	***B***	***SE B***	**β**	***B***	***SE B***	**β**	***B***	***SE B***	***B***
**Men**									
Age	0.384	0.049	0.256	0.086	0.072	0.057	0.100	0.072	0.066
Number of children	0.715**	0.492	0.048	0.936	0.486	0.062	1.083*	0.484	0.072
Education	0.689	0.749	0.031	0.428	0.708	0.020	0.339	0.704	0.015
Income	0.547	0.687	0.027	0.411	0.668	0.020	0.256	0.665	0.013
Duration of marriage				0.175*	0.081	0.095	0.146	0.081	0.079
Number of times divorced				5.611**	1.022	0.237	5.435**	1.016	0.230
Initiator Status: Participant vs Former Spouse				−3.997**	1.115	−0.139	−4.856**	1.133	−0.169
Initiator Status: Participant vs Mutual Agreement				2.402	1.407	0.066	1.180	1.437	0.032
New Partner Status: Both vs neither				−5.127*	2.327	−0.173	−5.088*	2.311	−0.172
New Partner Status: Both vs Participant Yes, Ex No				−1.723	2.617	−0.040	−1.666	2.599	−0.038
New Partner Status: Both vs Participant No, Ex Yes				−8.862**	2.486	−0.251	−8.341**	2.473	−0.236
Divorce Conflict							−0.337**	0.094	−0.117
*R*		0.27			0.43			0.44	
Adjusted *R*^2^		0.07			0.17			0.18	
*F*		16.49**			17.29**			17.15**	
Change *R*^2^					0.11			0.01	
*F* Change *R*^2^					16.57**			13.02**	
**Women**									
Age	−0.051	0.053	−0.033	−0.008	0.076	−0.005	0.011	0.076	0.007
Number of children	0.596	0.462	0.044	0.097	0.480	0.007	0.115	0.477	0.008
Education	0.087	0.700	0.004	−0.104	0.683	−0.005	−0.128	0.677	−0.007
Income	2.477**	0.752	0.118	2.254*	0.734	0.108	2.061**	0.729	0.098
Duration of marriage				0.021	0.080	0.013	0.024	0.079	0.015
Number of times divorced				−1.462	1.145	−0.051	−1.197	1.138	−0.042
Initiator Status: Participant vs Former Spouse				−5.617**	1.002	−0.202	−5.627**	0.994	−0.202
Initiator Status: Participant vs Mutual Agreement				0.125	1.361	0.003	−0.621	1.364	−0.016
New Partner Status: Both vs neither				−5.553**	2.021	−0.200	−5.898*	2.007	−0.212
New Partner Status: Both vs Participant Yes, Ex No				−0.904	2.459	−0.019	−1.156	2.440	−0.024
New Partner Status: Both vs Participant No, Ex Yes				−4.510*	2.192	−0.142	−3.941	2.179	−0.124
Divorce Conflict							−0.362**	0.093	−0.132
*R*		0.13			0.29			0.31	
Adjusted *R*^2^		0.01			0.07			0.09	
*F*		3.51*			7.13**			7.89**	
Change *R*^2^					0.07			0.02	
*F* Change *R*^2^					9.06**			15.06**	

*p < 0.05. **p < 0.001. When analyses were run with number of previous divorces coded as “1/2/3 or more,” the pattern of results remained the same. Moreover, when analyses were run with number of children coded as “1/2/3 or more,” the pattern of results also remained the same.

## Discussion

Pertaining to research question one, across gender, the study found that the mental health of Danish divorcees was significantly different from and worse than the Danish background population immediately following divorce. Further, across all mental health indicators, the magnitudes of these differences were large [i.e., Cohen’s (*d*) = 0.78–1.38]. The results for physical health were more equivocal. While both male and female divorcees reported better physical functioning in everyday life than the Danish background population, both genders also reported worse general health than the background population immediately following divorce.

The results for mental health corroborate existing research in the field and, notably, the effect sizes here were large, which may mainly reflect the timing of the collection of baseline data. With the unique opportunity to collect data very close to the juridical divorce (on average less than five days from juridical divorce) and the fact that the majority of the sample divorced without any prior separation period, data may have been less subject to a “time heals effect” (Hald et al., 2020a). Following Amato (2000) DSR, this means that time has not yet had a chance to mitigate the adverse effects of the divorce. Further, although caution needs to be taken regarding the generalizability of the sample, due to the non-probability sampling process, the results offer some of the first insights into *how* adverse the impacts of divorce on mental health may be immediately following divorce, using a range of common mental health indicators (Sander et al., 2020).

The equivocal findings concerning physical health among divorcees immediately following divorce, we speculate, mainly have to do with (a) the study sample, (b) the content of questions of the outcome measure, and (c) the timing of measurements. Accordingly, the study sample comprised relatively younger individuals as compared to the background population sample used for comparisons. The majority of the items from the physical health scale include responses to tasks most non-elderly individuals would easily be able to accomplish, but which may prove increasingly difficult with age (e.g., walking one block, dressing and bathing, or lifting or carrying groceries), and this may account for the better physical health among our study sample as compared to the background population. Further, as first suggested by Sander et al. (2020), when it comes to physical health, a “time hurts” effect may also be at play, whereby physical health is more adversely affected over the course of time following divorce than immediately after the divorce. A causal mechanism may be that reduced mental health increasingly adversely affects physical health over time (Sander et al., 2020). We encourage future studies to further investigate this.

From an applied point of view, across diverse samples and patient groups, better health-related quality of life as measured by the SF-36 has been found to be associated with lower risk of morbidity, mortality, cancer as well as the recurrence of cancer, anxiety, and depressive symptoms (e.g., Lacson et al., 2010; Saquib et al., 2011; Folker et al., 2019). Further, multiple studies have found that worse health-related quality of life as measured by the SF-36 instrument is predictive of higher occurrence of work absence due to sickness, hospitalizations, and higher health care costs among both general populations and across multiple subpopulations (e.g., Lacson et al., 2010; Laaksonen et al., 2011; Pymont and Butterworth, 2015). In conjunction with the study results, especially for mental health, this means that there is sound human and financial reasoning in developing interventions that may help divorcees cope with adverse (mental) health effects of their divorce and, that among many divorcees, the need for help may be especially pronounced immediate following their divorce.

Pertaining to research question 2 and the study hypothesis, it was found that for men, lower age and higher income added to the prediction of better physical health. Among women, higher income, fewer previous divorces, new partner status, and lower levels of divorce conflict added to the prediction of better physical health. For mental health, among men, it was found that more children, more previous divorces, participant divorce initiation, new partner status, and lower levels of divorce conflict added to the prediction of better mental health, while for women, higher income, participant divorce initiation, new partner status, and lower levels of divorce conflict were found to add to better mental health. Moreover, our study hypothesis that divorce conflict would add to the overall prediction of mental health, even when other sociodemographic variables and divorce characteristics were controlled for, was supported. Of note, lower divorce conflict also predicted better physical health for women.

The current study indicates that, already at the time of or close to juridical divorce, higher degrees of divorce conflict are associated with worse mental health, even after accounting for other sociodemographic variables and divorce-related factors. This may not be surprising, given that higher degrees of divorce conflict are likely to negatively interfere with or complicate important decisions and life choices around the time of juridical divorce, like division of property, co-parenting, and child custody. This study finding accentuates the need to focus on divorce conflict levels already at divorce onset (Hald et al., 2020d).

Amato’s DSR theory stipulates that the adverse effects of divorce depend on the interplay between risk and protective factors (Amato, 2010). These factors include many of those found in this study to significantly predict both mental and physical health, including income (DSR = economic security, standards of living), new partner status (DSR = having a new partner), and levels of divorce conflict (DSR = conflict with the former partner). Accordingly, the results of this study may be seen as support for Amato’s DSR theory, in that DSR theory views divorce “not as a discrete event, but as a process that unfolds over months and even years” (Amato, 2010, p. 10). Moreover, it follows that mental and physical health may already be adversely affected prior to the juridical divorce as a consequence of a prolonged stressful and/or unsatisfactory relationship (Hald et al., 2020c). Therefore, the measurements of mental and physical health employed in this study, done immediately after juridical divorce with little or no prior separation period, may “capture” the mental and physical health consequences of this “…process that unfolds over months and even years” (Amato, 2010, p. 10).

Notably, even in an egalitarian society such as the Danish one, with a large public sector, a well-developed welfare system, and fewer differences between rich and poor as compared to most other Western countries, higher income still significantly predicted mental well-being among women and physical well-being among both men and women. In accordance with DSR theory, this suggests that income may be a key protective factor against negative divorce-related health impacts (Leopold, 2018), even in highly egalitarian societies. Even more so, income may be more important than level of education, a variable previously found to be related to post-divorce psychological and physical health outcomes (Cohen and Finzi-Dottan, 2012; Perrig-Chiello et al., 2015), but which was not found to significantly predict mental or physical well-being in this study.

To the best of our knowledge, this study is the first to include a large sample of very recently divorced individuals, employ standardized and validated mental and physical health measures consisting of multiple health-related indicators with available background population data for direct comparisons, and a multitude of sociodemographical and divorce-related variables previously shown to be associated with health-related outcomes. However, when evaluating the results, the following study limitations should be taken into consideration. The study used a non-probability sample of divorcees and employed self-report measures, which may limit the generalizability of findings. Specifically, the study sample may have consisted of individuals with more conflicts and more mental and physical problems than those who did not participate in the study, as these individuals may have believed that the intervention platform would be particularly helpful to them. Conversely, it may also be that people with more conflicts and more mental and physical problems may have decided not to participate because it may have felt threatening to their sense of self (Howell and Shepperd, 2012; DiBello et al., 2015), and thus, are underrepresented in the current study. Additionally, we were unable to determine if both partners in a prior marriage participated in the study, which may affect the assumption of independence of data in the analyses. Further, due to the cross-sectional nature of our data, the results preclude causal inferences. Lastly, while the Danish context is interesting for several reasons, including the minimal societal stigma surrounding divorce and the presence of greater gender and income equality, there is also great acceptance of non-marital cohabitation, such that many couples choose to not get legally married. As the study targeted formerly legally married individuals, individuals who cohabitate were not recruited, and thus, it is unclear whether the study results may generalize to this group of individuals. However, we expect that the relationship dissolution process is similar for married and cohabitating individuals, to the extent that there can be children involved and shared assets (e.g., house). Therefore, we do not have reason to expect that non-married individuals differ from married individuals; however, future research should seek to examine this point.

In conclusion, the study found that the health-related quality of life of Danish divorcees immediately following divorce was significantly different from and worse than the comparative Danish background population. Further, higher levels of divorce conflict predicted worse mental health even after controlling for other sociodemographic variables and divorce characteristics often targeted in research on the interplay between divorce and health. The findings underscore the relevance of providing divorce interventions for divorcees as early as possible following their divorce to improve health-related quality of life.

## Data Availability Statement

The raw data supporting the conclusions of this article will be made available by the authors, without undue reservation.

## Ethics Statement

The studies involving human participants were reviewed and approved by the Danish Data Protection Agency and the Regional Scientific Ethical Committee of Copenhagen, Denmark. The patients/participants provided their written informed consent to participate in this study.

## Author Contributions

This original research report is part of the doctoral thesis for SS. SS and GH were responsible for the design of the intervention and the study protocol and also responsible for the manuscript writing. JS was responsible for data analysis. CØ and AC were responsible for feedback and editing. All authors have read and approved the final manuscript.

## Conflict of Interest

For due diligence, we would like to declare that the University of Copenhagen, Denmark, where the authors work, owns the digital intervention platform “Cooperation after Divorce (CAD)” while two of the co-authors (GH and SS) hold the commercial license and intellectual property rights to the platform through the Company “CAD” (Samarbejde Efter Skilsmisse ApS). The reviewer LL declared a shared affiliation, with no collaboration, with the author to the handling editor at the time of the review.
